# Evolutionary Dynamics of the Pericentromeric Heterochromatin in *Drosophila virilis* and Related Species

**DOI:** 10.3390/genes12020175

**Published:** 2021-01-27

**Authors:** Alexander P. Rezvykh, Sergei Yu. Funikov, Lyudmila A. Protsenko, Dina A. Kulikova, Elena S. Zelentsova, Lyubov N. Chuvakova, Justin P. Blumenstiel, Michael B. Evgen’ev

**Affiliations:** 1 Engelhardt Institute of Molecular Biology of Russian Academy of Sciences, 119991 Moscow, Russia; aprezvykh@yandex.ru (A.P.R.); sergeifunikov@mail.ru (S.Y.F.); protsenko.la@phystech.edu (L.A.P.); zelentsova2007@yandex.ru (E.S.Z.); lyubov.astakhova@gmail.com (L.N.C.); 2 Moscow Institute of Physics and Technology, 117303 Dolgoprudny, Russia; 3 Koltzov Institute of Developmental Biology of Russian Academy of Sciences, 119334 Moscow, Russia; dinakulikova@mail.ru; 4 Department of Ecology & Evolutionary Biology, University of Kansas, Lawrence, KS 66045, USA; jblumens@ku.edu

**Keywords:** Drosophila, heterochromatin, gene expression, molecular evolution, transposable elements, piRNAs

## Abstract

Pericentromeric heterochromatin in *Drosophila* generally consists of repetitive DNA, forming the environment associated with gene silencing. Despite the expanding knowledge of the impact of transposable elements (TEs) on the host genome, little is known about the evolution of pericentromeric heterochromatin, its structural composition, and age. During the evolution of the *Drosophilidae*, hundreds of genes have become embedded within pericentromeric regions yet retained activity. We investigated a pericentromeric heterochromatin fragment found in *D. virilis* and related species, describing the evolution of genes in this region and the age of TE invasion. Regardless of the heterochromatic environment, the amino acid composition of the genes is under purifying selection. However, the selective pressure affects parts of genes in varying degrees, resulting in expansion of gene introns due to TEs invasion. According to the divergence of TEs, the pericentromeric heterochromatin of the species of virilis group began to form more than 20 million years ago by invasions of retroelements, miniature inverted repeat transposable elements (MITEs), and Helitrons. Importantly, invasions into the heterochromatin continue to occur by TEs that fall under the scope of piRNA silencing. Thus, the pericentromeric heterochromatin, in spite of its ability to induce silencing, has the means for being dynamic, incorporating the regions of active transcription.

## 1. Introduction

Large segments of the eukaryotic genome are packed into two basic forms of chromatin termed euchromatin and heterochromatin [1]. Euchromatic regions comprise most of the length of the chromosomes and are highly enriched with actively-transcribed loci, including protein-coding genes [2]. Heterochromatin is largely concentrated at pericentromeric and telomeric chromosomal domains termed constitutive heterochromatin [1,2]. Constitutive heterochromatin tends to be late replicating and shows low frequency of meiotic recombination [3]. These genomic segments are dominated by repetitive DNA sequences (up to 80%), including tandem repeats or satellite DNA as well as remnants of diverse transposable elements (TEs) [1,2]. A distinctive property of heterochromatin in *Drosophila* is its enrichment with di- or trimethylated H3K9 and heterochromatin protein 1 (HP1a) [4,5]. Functionally, heterochromatic domains play a critical role in chromosome segregation, telomere protection, and suppression of TEs activity [1]. Due to its highly repetitive nature often comprising millions of base pairs, assembly and annotation of pericentromeric heterochromatin remains challenging and is often left out of the picture in most genome-wide studies, especially for those that include non-model organisms [6,7,8,9,10]. Despite the fact that our understanding of heterochromatin structure, its dynamics, and function has grown over the last decade, little is known about the evolution of pericentromeric heterochromatin, its age, and structural differences in related species.

Although gene-poor, constitutive heterochromatin is not devoid of genes, frequently forming islands of active transcription that are dependent on the local environment and particular *cis* and *trans*-acting elements for normal expression [11,12,13,14]. Despite the repressive environment, hundreds of active genes have been identified in the pericentric heterochromatin of *D. melanogaster* [15]. Interestingly, in the course of evolution, due to chromosomal inversions or transposition events the same genes can be embedded in distinct genomic loci, either euchromatic or heterochromatic, in different related species [16]. For example, two genes, *light* and *Yeti*, located in pericentric region in *D. melanogaster*, reside in a euchromatic region in *D. virilis* on the same chromosomal elements [12,17].

Here we performed a comprehensive analysis of a large fragment of the pericentromeric heterochromatin in *D. virilis* and related species, focusing on molecular evolution of repetitive DNA and structural genes embedded in this region and the age of various TEs invasions. All studied genes (*Stim*, *CG8578*, *CG33172*, *Myb*, and *Ranbp16*) are located in euchromatic loci of the *D. melanogaster*, while in other studied *Drosophila* species belonging to Sophophora and Drosophila subgenus they are found in genomic regions with a high density of repetitive DNA elements upstream, downstream and within introns, suggesting their location in constitutive heterochromatin. Regardless of the euchromatic or heterochromatic surroundings, the amino acid composition of all the genes remains largely unchanged, keeping the protein-coding regions under purifying selection in the *Drosophilidae* lineage. However, in the course of evolution the selective pressure affects different parts of genes in varying degrees. Herein, we show that *Myb* and *CG33172* genes retained their organization in all analyzed species, regardless of their euchromatic or heterochromatic environment. On the other hand, the structure of *CG8578* and *Ranbp16* genes was dramatically changed due to an increase in their intron size by numerous transposable element insertions. 

According to TEs substitution levels, the current state of the pericentric heterochromatin in the species of virilis group began to form more than 20 million years ago by invasions of retroelements predominantly belonging to LTR/Gypsy and LINE/R1 families, followed by the occupation by miniature inverted repeat transposable elements (MITEs) and Helitrons. Interestingly, invasions of TEs into the pericentromeric region apparently continued to occur by retroelements of both LINE and LTR-containing classes that fall under the regulation of piRNA machinery. 

## 2. Materials and Methods

### 2.1. Drosophila Genomes and Sequence Analyses

*Drosophila* genomes and gene sequences for comparative analysis were extracted from FlyBase and NCBI databases. In the case of absence or incomplete gene annotation (e.g., for *D. pseudoobscura* and *D. novamexicana*), orthologs were retrieved with TblastN using protein sequence of the most closely related species (i.e., *D. persimilis* for *D. pseudoobscura* and *D. virilis* for *D. novamexicana* and other virilis group species) [18]. All the query subjects mapped to the same DNA strand adjacent to each other with E-value of 1E^e-80^ were considered as true and used for reconstruction of coding sequences. Sequences between mapped query subjects were considered as introns. All essential information, including genes IDs, genomes IDs, and genomic coordinates of genes *Stim*, *CG8578*, *CG33172*, *Myb*, and *Ranbp16* in all studied species, is listed in Table S1. Orthologous sequences of genes for *Anopheles gambiae* were extracted from VectorBase (https://www.vectorbase.org/). Protein sequences of mouse and human were extracted from UniProt (https://www.uniprot.org/). Protein motifs were scanned using the PROSITE database and methodology [19,20].

Multiple sequence alignment was performed with ClustalW and Clustal Omega programs (https://www.ebi.ac.uk/Tools/msa/) for nucleotide and amino acid alignments, respectively. Multiple protein alignments were visualized with Jalview [21]. 

Exon-intron structure of studied genes were visualized using WormWeb exon-intron graphic maker (http://wormweb.org/exonintron). Visualization of blast results was done with Kablammo [22]. 

### 2.2. Transposable Elements Annotation and Analysis

Custom libraries of TEs were used to mask genome and genomic regions of studied species. For *D. melanogaster* we used the canonical sequences of transposable elements fetched from http://www.fruitfly.org/p_disrupt/TE.html, for *D. yakuba*, *D. persimilis*, and *D. mojavensis* we applied computationally predicted libraries of TEs generated with REaS that are available at FlyBase (ftp://ftp.flybase.net/genomes/aaa/transposable_elements/ReAS/v2/consensus_fasta/), for *D. virilis* the combined libraries of annotated and computationally predicted TEs were used [23,24]. Finally, for *D. novamexicana* we made de novo annotation of TEs by RepeatModeler2 software [25]. MITE-Tracker was used to expand the canonical TE library with sequences of miniature inverted repeat transposable elements (MITEs) [26]. In addition, recently described DAIBAM MITE (GenBank: EU280326) was added to MITEs [27]. In order to remove redundant sequences annotated by RepeatModeler2 and MITE-Tracker, we performed filtration of libraries from rRNA and mitochondrial DNA. We also discarded shorter sequences that blast to longer ones using CD-HIT (https://github.com/weizhongli/cdhit) and removed sequences shorter than the ORF of the smallest known eukaryotic transposase in Repbase (Ac-type transposase homolog (*Glomus mosseae*)—76 amino acids). 

To screen genomes and genomic regions for TEs, we used RepeatMasker (v 4.1.0) in RM-BLAST mode, with “-nolow” option focusing only on TEs of custom libraries without masking low-complexity and simple repeats [28]. This masking method of TE sequences gives us information about the localization of each TE invasion, as well as nucleotide substitution level, insertions, deletions, and DNA strand. To determine the Kimura 2-distance of repeat sequences in the genomic regions, we used script ‘calcDivergenceFromAlign.pl’ from the RepeatMasker package.

Visualization was performed using ggplot2 in R environment [29,30]. 

### 2.3. DNA Extraction, Sequencing, and Assembly of the Genome

High molecular weight DNA extractions were performed with the Blood Cell and Culture Midi Kit (Qiagen, Germantown, MD, USA) based on Chakraborty et al. with modifications [31]. Approximately 150 wandering third-instar larvae (~500 mg) of *D. virilis* strain *9* were collected and rinsed in distilled water, then immediately frozen in liquid nitrogen and ground to powder. The mortar was pre-chilled with liquid nitrogen prior to grinding and the resulting powder was directly transferred into Buffer G2. DNA extraction was performed across 5 columns, using a total of 47.5 mL G2, 380 μL RNAse A (10 mg/mL) and 1 mL of Protease (~1 AU/mL) from the Qiagen Kit. This volume was then placed in a 50 °C shaker overnight. After overnight incubation, debris was spun down and poured evenly across 5 columns. The elution was performed according to manufacturer’s instructions, precipitated with 0.7 volumes of isopropanol and resuspended in 30 μL EB Buffer. Yield was lower than expected. The final DNA concentration was estimated with a Qubit yielding approximately 2 μg of high molecular weight DNA. 

Preparation of the library was performed using the SQK-RAD002 Rapid Kit (ONT, Oxford, United Kingdom) following the recommended protocol from ONT using 250 ng of DNA. Sequencing was performed on a MinION (ONT) with a FLO-MIN-106 R9 flow-cell (ONT) and basecalling was performed by Albacore algorithm implemented in the MinKNOW platform in July 2017.

Genome assembly of *D. virilis* strain *9* was performed by Flye genome assembler using Nanopore reads with N50 = 22,767 bp and estimated coverage 70× [32]. Draft genome assembly consisted of 564 contigs with total length = 182.3 Mb and N50 = 18.8 Mb. Error correction of draft assembly was performed by 4 iterations of Pilon using Illumina paired-end reads fetched from short read archive (SRA; SRX496597) [33]. Quality and completeness of the final polished assembly were validated by BUSCO software using BuscoDB version 4 with Diptera lineage [34]. The final percent of complete and single-copy orthologs equals 96.5%, representing high-quality and complete genomic assembly of strain *9* which was deposited into NCBI under the accession number Bioproject PRJNA687501. 

### 2.4. ChIP-seq, RNA-seq, and Small RNA-seq Analyses

Raw data of genome binding/occupancy (ChIP-seq), transcriptome (RNA-seq) and small RNA (small RNA-seq) profiling for *D. virilis* strains *160* and *9* were obtained from GEO and SRA and used in the analyses. They include: GSE59965—contains data for strain *160* including ovarian RNA-seq and ChIP-seq of H3K9me3; SRP060632- contains ovarian small RNA-seq data for strains *160* and *9*. 

For analysis of *D. virilis,* we used the reference genome sequence (release r.1.06 from FlyBase), assembly of *160* (ASM798932v2 from NCBI) and our assembly of strain *9*. Prior to mapping, all raw reads were subjected to adapter clipping, filtering by quality (80% of nt must have at least 20 Phred quality) and by length (>20 nt) using TrimGalore (https://github.com/FelixKrueger/TrimGalore). Small RNA-seq reads were further subjected to subtraction of reads matching all rRNA, tRNA, snRNA and miRNA sequences of *D. virilis*. Additionally, we selected only 23–39 nt reads from small RNA-seq data and considered them as piRNAs. Then, sequences were aligned to the corresponding *D. virilis* genomes (refgen, 160 and 9) using Bowtie with the following settings: “-m 1 -v 0”, retaining only uniquely aligned reads with zero mismatches [35]. Output sequence alignment map (SAM) files were converted to binary (BAM) format using SAMtools [36]. BAM files were converted to wiggle (WIG) format using bamToWig script (https://github.com/craiglowe/bamToWig). Aligned reads in wig format were visualized using the Integrative Genome Viewer (IGV) [37]. 

### 2.5. Testing for Selection

Ratio of nonsynonymous to synonymous substitutions (dN/dS) was estimated using PAL2NAL software by converting multiple sequence alignment of proteins and the corresponding nucleotide sequences into a codon alignment, and the calculation of synonymous (dS) and non-synonymous (dN) substitution rates using codeml program implemented in PAML package [38,39]. FEL (Fixed Effects Likelihood) algorithm was used to estimate selection on a per-site basis [40].

### 2.6. Cytology and DNA In Situ Hybridization

Two days before dissection, *D. virilis* larvae were grown at 18 °C on standard medium supplemented with live yeast. Salivary glands from 3rd instar larvae were dissected in 45% acetic acid and squashed. DNA probes corresponding to *D. virilis Myb* (Dvir\GJ18431; FlyBase ID: FBgn0205590) were prepared by PCR using gene-specific primers (Forward_GCAAGTGCGAGCACTGAAAA; Reverse_TGCATACTGAGGTGTGCCAG). Then, DNA probe was biotinylated by nick translation using Biotin-14-dATP (Thermo Fisher Scientific, Waltham, MA, USA) as described in [41]. Localization of the probe was made using the cytological map of *D. virilis* chromosomes [42]. Images were obtained with binocular microscope Nikon Alphaphot-2 YS2 (Japan).

## 3. Results and Discussion

### 3.1. Highly Repetitive Region of Pericentromeric Heterochromatin of X Chromosome of D. virilis Incorporates Several Actively-Transcribed Protein-Coding Genes 

**Figure 1:**
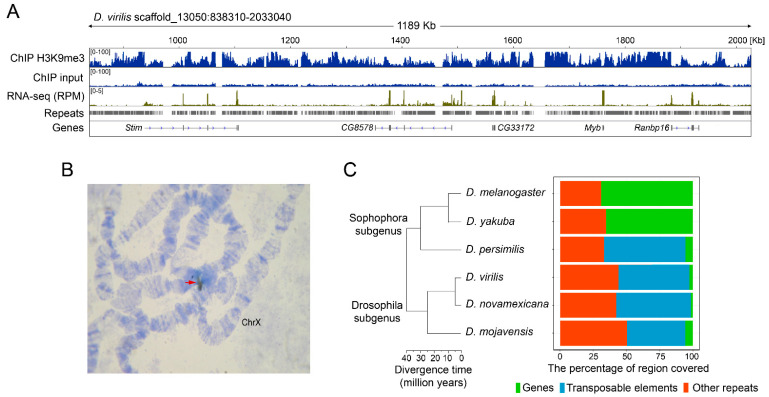
Landscape of pericentric heterochromatin region in chromosome X of *D. virilis*. (**A**) Genomic map of the region in scaffold_13050 of reference genome of *D. virilis* defined by protein-coding genes *Stim*, *CG8578*, *CG33172*, *Myb*, and *Ranbp16* located in this scaffold with mapped ChIP-seq reads of heterochromatic markers H3K9me3. Input for ChIP-seq data is also shown. RNA-seq reads were normalized to the sequence depth (RPM, reads per million). Repeats and protein-coding genes are shown as different tracks. (**B**) DNA in situ hybridization of *Myb* gene probe to polytene chromosomes of *D. virilis*. The black arrow indicates the hybridization signal at the base of chromosome X of *D. virilis*. (**C**) Genomic content between the same five protein-coding genes *Stim*, *CG8578*, *CG33172*, *Myb*, and *Ranbp16* in different *Drosophila* species. Due to different distances between genes, the number of annotated genomic features was normalized to the length of the analyzed region for each species. Transposable elements include LTR and non-LTR (LINE) retrotransposons, DNA, miniature inverted repeat transposable elements (MITEs), and Rolling circle elements. Other repeats represent non-transposable element (TE) related sequences including satellite DNA and simple repeats. Unrooted phylogenetic tree indicates the relationships among species with the estimated time of divergence according to Clark et al. and O’Grady et al. for the virilis group [43,45].

In order to obtain insight into the structural composition of pericentromeric heterochromatin of *D. virilis,* we searched for assembled contigs in the genome of *D. virilis* sequenced by the 12 Drosophila genomes consortium that would be highly enriched with the heterochromatic mark H3K9me3 [43]. The performed analysis revealed one of such genomic segments in the genome of *D. virilis* (scaffold_13050; genome release r1.06). This scaffold is highly enriched with H3K9me3 and includes five protein-coding genes (*Stim*, *CG8578*, *CG33172*, *Myb*, and *Ranbp16*) present in single copies in the genome and having orthologs in *D. melanogaster* (Figure 1A). The rest of the scaffold sequence comprises hundreds of copies of various repeats that surround the gene loci and occupy their introns (Figure 1A). To confirm that this scaffold resides in a pericentromeric heterochromatic region in *D. virilis*, we performed DNA in situ hybridization on polytene chromosomes using the unique sequence of one of the genes in this scaffold (*Myb* gene) as a probe. The performed analysis indicated that *Myb* is located at the base of chromosome X near the chromocenter in *D. virilis,* which is apparently a part of the pericentric heterochromatin (Figure 1B). A distinctive well-known feature of heterochromatin is the ability to silence euchromatic genes placed within the heterochromatic environment, a phenomenon called position effect variegation (PEV) [2,44]. In spite of the silencing effect of PEV, these protein-coding genes are actively transcribed, according to the RNA-seq mapping profile which shows expression predominantly of the exonic regions of the genes (Figure 1A). 

Given that repositioning of genes from euchromatin to heterochromatin during genome evolution is not unusual in the *Drosophilidae* lineage, we analyzed the genomic environment flanking all five aforementioned genes (20 Kb upstream and downstream from the genes coordinates) in *Drosophila* species separated by evolutionary distances from 5 to 40 million years [43]. These species include representatives of the melanogaster group (*D. melanogaster* and *D. yakuba*) and the obscura group (*D. persimilis*), with both groups belonging to the Sophophora subgenus. In addition, we investigated species of the virilis group (*D. virilis* and *D. novamexicana*) and the representative of the repleta group (*D. mojavensis*) that belong to the Drosophila subgenus (Figure 1C, Table S1). The analysis shows that while genes *Stim*, *CG8578*, *CG33172*, *Myb*, and *Ranbp16* in the species of melanogaster group are embedded within a large gene cluster (regions consist of structural genes for up to 70%), their orthologs in other *Drosophila* species reside in the genomic regions mostly occupied by repetitive sequences (genes comprise less than 10% of the sequence) (Figure 1C). Thus, one may conclude that genes *Stim*, *CG8578*, *CG33172*, *Myb*, and *Ranbp16* in the species belonging to the melanogaster group are located in euchromatin, in the region containing a large gene cluster. On the other hand, the localization of the studied genes in the environment packed with repetitive elements and apparently representing a pericentric heterochromatic region is typical for most other *Drosophila* species studied here. 

### 3.2. All Studied Heterochromatic Genes of D. virilis Are Under Purifying Selection

Next, we examined whether coding sequences of *Stim*, *CG8578*, *CG33172*, *Myb*, and *Ranbp16* underwent negative (purifying) or positive selection during evolution and what was the impact of heterochromatic location on the molecular evolution of these genes. It should be noted that all studied genes are present as single copies in the genomes and we have not observed any evidence of their duplication and pseudogenization process. To this end, we calculated the ratio of non-synonymous to synonymous substitutions (dN/dS) (Figure S1) using representatives of the Sophophora and Drosophila subgenera, as well as the sequences from *Anopheles gambiae* as an outgroup. The results suggest that all studied genes are under purifying (negative) selection (dN/dS < 0.2 in all pairs of comparison) when present either in heterochromatin or euchromatin (Figure S1A). Given that the strength of natural selection may have changed in these genes at the amino acid sequence level we performed a calculation of the ratio of nonsynonymous to synonymous substitutions at each site using a fixed effects likelihood (FEL) method [40]. This analysis showed that most of the sites of these genes are under pervasive negative (purifying) selection (159 of 305 in *CG8578*, 645 of 967 in *Ranbp16*, 349 of 535 in *Stim*, 291 of 514 in *Myb* and 255 of 901 in *CG33172*, *p* < 0.1; Figure S1B). We found only a few sites under the pressure of pervasive positive selection at our specified threshold (*p* < 0.1, 8 of 901 sites in *CG33172*, 2 of 514 in *Myb* and 1 of 535 in *Stim*; Figure S1B). 

**Figure 2:**
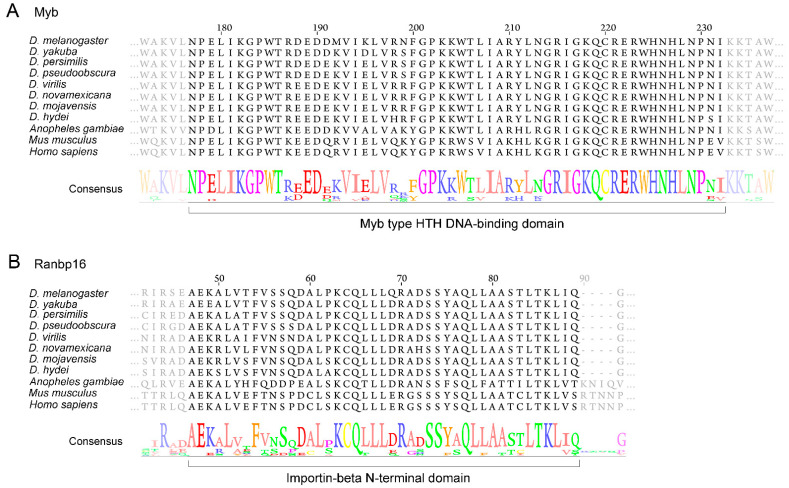
Multiple protein alignment of Myb type Helix-Turn-Helix (HTH) DNA-binding domain of Myb protein (**A**) and Importin-β domain of Ranbp16 protein (**B**) across selected taxa.

We also performed multiple alignments of protein sequences of Myb, Ranbp16, CG8578 (Drosophila homolog of LRRFIP2 (LRR binding FLII interacting protein 2)), and CG33172 (Drosophila homolog of WDR6 (WD repeat domain 6)) from Diptera species and their orthologs in mouse and human. The results show a high degree of conservation of both proteins even between dipteran and mammalian species (Figure 2 and Figure S2). The consensus sequences of aligned proteins are properly recognized as Myb type Helix-Turn-Helix (HTH) DNA-binding domain, Importin-β N-terminal domain, leucine-rich repeat flightless-interacting protein domain, and WD40 repeat domain that characterize the described structure of Myb, Ranbp16, CG8578, and CG33172 proteins, respectively (Figure 2 and Figure S2).

### 3.3. Evolutionary Changes in the Organization of Myb, Ranbp16, CG8578, and CG33172 Genes in Drosophila Species

During speciation, the genomes of *Drosophila* species underwent multiple chromosome rearrangements that disrupted gene order, either modifying or preserving their function [16,46,47]. Despite the contrasting chromatin structure and local repressive environment of heterochromatic regions enriched with repetitive DNA, all studied genes were shown to be under purifying selection due to their highly conserved and essential function. However, the overall gene organization may not be as conserved as the protein-coding region of genes located in heterochromatin. It was reported in a number of studies, that genes located in the heterochromatic environment may increase their size due to multiple TE insertions in their introns [12,13,17,48]. To study how the structure of the genes juxtaposed in the heterochromatic regions has been changed in the course of evolution, we compared the structure of these genes in diverse *Drosophila* species. 

First, we retrieved the available sequences of *Myb*, *Ranbp16*, *CG8578*, and *CG33172* genes from *D. melanogaster*, *D. yakuba*, *D. persimilis*, *D. pseudoobscura*, *D. virilis*, *D. novamexicana*, *D. mojavensis*, and *D. hydei* from FlyBase and NCBI databases. Due to the missing or incomplete gene annotation, the sequences of genes for *D. pseudoobscura* and *D. novamexicana* were reconstructed using the sequences of related *D. persimilis* and *D. virilis*, respectively, using TblastN algorithm [18]. Gaps in the continuous alignment were considered as introns. To date, unfortunately, the full annotation of genes including 5′ and 3′UTR (untranslated region) of most *Drosophila* species, except for *D. melanogaster*, is absent. To resolve this issue and obtain the most complete sequence of studied genes, we used sequences from closely related species (for example, *D. persimilis* sequences were used to resolve the *D. pseudoobscura* gene structure, etc.). Only those alignments that mapped to the same DNA strand adjacent to the existing annotated sequence with E-value > e^−80^ were considered as relevant and used for reconstruction of gene structure and comparative analysis. All information, including accession numbers and genomic coordinates of the studied genes in *Drosophila* species, is listed in Table S1.

**Figure 3:**
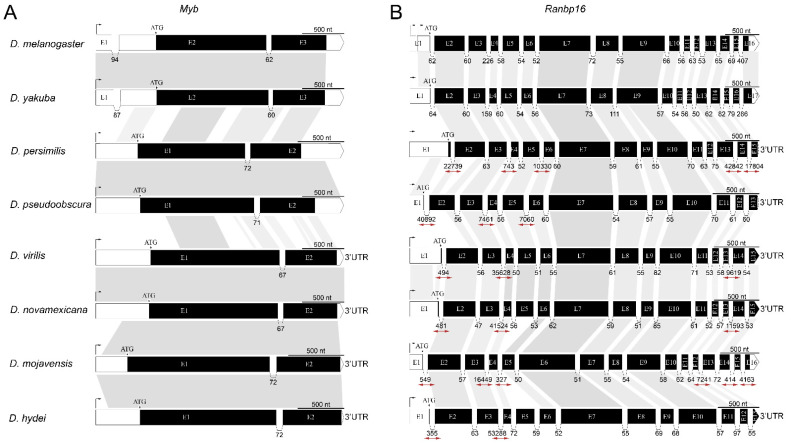
Comparative gene organization of *Myb* (**A**) and *Ranbp16* (**B**) genes among eight *Drosophila* species. White and black regions on the scheme denote untranslated and coding regions, respectively. The grey area between genes indicates homologous regions defined using nucleotide BLAST basing on the percentage of identity not less than 70. Homology of introns is shown only for the *Myb* gene. The rightwards corner arrow at the beginning of genes indicates the transcriptional start site. Due to the large length of some introns in most *Drosophila* species, all introns of *Ranbp16* are shown not in their real sizes. Numbers underneath the introns reflect their size in nucleotides. Introns that were increased in size by transposable element insertions are highlighted by red bidirectional arrows. The 3′untranslated regions (3′UTR) are not given for most of the genes due to the absence of their annotations and homology to those from closely related species.

During the evolution, the structure of *Myb* and *CG33172* genes was preserved virtually unchanged in terms of overall gene length and exon-intron organization (Figure 3A and Figure S3A). A single intron located in the protein-coding region of *Myb* is conserved both in position and approximate size in the species of both subgenera (Figure 3A). However, sequence homology of this intron is conserved only within species belonging to one subgenus, Sophophora or Drosophila. This suggests that it is not the intron sequence itself, but rather its length that is likely important for gene expression. Therefore, the heterochromatic *Myb* gene tends to be stable during the evolution of the Drosophila genus and retains the single intron of approximately the same size in different species (Figure 3A). This type of organization is also applied to *CG33172,* which preserved the overall exon-intron organization during the evolution of the *Drosophilidae* lineage (Figure S3A). However, in the virilis species group, the second intron of *CG33172* was enlarged due to the insertion of transposons of DNA/P family (Figure S3A). 

In contrast to Myb, the structure of Ranbp16 and CG8578 is more complex and demonstrates greater flexibility mostly due to the extension of total gene length by increasing the size of its introns (Figure 3B and Figure S3B). The length of Ranbp16 gene in the species of melanogaster group (*D. melanogaster* and *D. yakuba*) is ~6700 bp and includes up to 15 introns not exceeding 407 bp in length. In the course of evolution, the gene structure of Ranbp16 in other studied Drosophila species was expanded to varying degrees by multiple insertions of TEs. For example, the length of Ranbp16 gene is approximately 100 Kb in *D. obscura*, 55 Kb in *D. virilis* and 40 Kb in *D. mojavensis* (Figure 3B, Table S1). For CG8578 the largest intronic expansion is observed for *D. persimilis* (~80 Kb), *D. mojavensis* (~88 Kb) and virilis group species (~137 Kb in *D. virilis* and ~74 Kb in *D. novamexicana*), while the length of this gene in *D. melanogaster* is only 3.4 Kb (Figure S3B, Table S1). Interestingly, the introns prone to TE insertions increasing the length of the gene are strictly defined. They mostly include the first ones from the transcriptional start site as well as a few introns at the distal end of the gene (Figure 3B and Figure S3B). The extension of other introns located in the middle of the gene was not observed in any of the studied species, suggesting that these introns may have a structural or regulatory role in the gene function.

Despite strong purifying selection acting on these genes, the homology of exon sequences is higher within a subgenus and closely-related species, As indicated in Figure 3 and Figure S3 nucleotide conservation is much lower between Sophophora and Drosophila subgenera in comparison with the species within subgenus, e.g., *D. melanogaster* and *D. yakuba* or *D. virilis* and *D. novamexicana* (Figure 3 and Figure S3). Notably, even whole coding exons may have no homology at the DNA level between two related species *D. mojavensis* (exon 6) and *D. hydei* (exon 5) as indicated for CG8578 gene in Figure S3. However, as we showed previously, amino acid sequences exhibit a high degree of conservation suggesting that changes occurred mostly at the nucleotide level and resulted in synonymous substitutions (Figures S1 and S2). 

The adaptive value of different intron lengths in the same gene in related species is unknown. A simple explanation of different intron content of the studied genes (Myb vs. Ranbp16) stems from the fact that selective pressure favors short introns in highly expressed genes rather than in genes that are expressed at lower levels [49]. In addition, a phenomenon called intron delay may limit the ability of cells with a short mitotic cycle to transcribe long primary transcripts [50,51]. Thus, Myb, which is a highly expressed gene throughout the Drosophila development, retained its organization regardless of its euchromatic or heterochromatic location. On the other hand, Ranbp16 and CG8578 are expressed at a low level and probably exhibit tissue-specific patterns of expression. This may result in their more labile organization on the evolutionary timescale, with a remarkable increase in size in the heterochromatin context.

An unexplained question is the differential susceptibility of introns to expansion due to TEs insertions. A dramatic increase in Ranbp16 and CG8578 gene length in several species occurred only due to the expansion of some but not all introns spanning the gene. It may be thought that selective pressure affects different parts of genes to varying degrees, enabling the expansion of some introns and preserving the length of others. In genomes of animals, plants, fungi, and protists, intron positions could be conserved throughout prolonged evolutionary times, suggesting their involvement in gene expression and regulation, including mRNA processing [52,53,54]. Numerous studies performed on animals and plants have demonstrated that introns may carry functional cis-acting elements affecting gene expression even in the absence of promoter sequences [55,56,57,58,59]. Recently, it was shown that nucleosomes preferentially occupy exons rather than introns, and this phenomenon seems to be interconnected with specific histone modifications favoring gene expression [60,61,62]. Thus, conservation of specific introns in the evolution of heterochromatin located genes suggests their role in regulation of expression of these genes. However, the cross-talk between regulatory elements in introns, gene architecture, chromatin structure, and nucleosome positioning and modifications, especially in the heterochromatin, should be studied in more detail.

### 3.4. Pericentromeric Heterochromatin Regions Demonstrate Similar Composition of TEs but Differ Quantitatively in Their Content in the Virilis Phylad

To determine TEs composition and age of the pericentromeric heterochromatin in *D. virilis* and closely-related species, we extended our analysis to *D. virilis* strains from different sources. In addition to the reference genome, we included in the analysis the laboratory strain 160 with recently released genome assembly (NCBI #ASM798932v2) [63]. Additionally, to analyze intraspecific peculiarities of the structural composition of pericentric heterochromatin we performed DNA sequencing of wild-type strain 9 (Batumi, Georgia) using Oxford Nanopore technology followed by its de novo assembly (see Materials and Methods). Notably, these two *D. virilis* strains are of special interest because these strains have been important in demonstrating a role for RNA silencing by PIWI-interacting RNAs (piRNAs) in protecting the genome against TE activation in the germline and hybrid sterility, a phenomenon known as hybrid dysgenesis [64,65]. Finally, to characterize interspecific differences the genome of *D. novamexicana* was analyzed as well. Given that the genomes of strain 9 and *D. novamexicana* were not assembled to the chromosomal level, to retrieve a comparable heterochromatin fragment we mapped Stim, CG8578, CG33172, Myb, and Ranbp16 onto the genome and extracted all contigs with good alignments (E-value not less than 1E^−80^). At the end, we combined these fragments together to obtain a comparable fragment. In the case of the reference strain and the genome of strain 160, all the genes were found on the single contig. Thus, a comparable region was selected using the flanking genes (Stim on the left and Ranbp16 on the right) by adding 100 Kb up and downstream from them. The resulting heterochromatic region was ~1.2 Mb in the reference genome (Figure 1A), the fragment of the same length in strain 160, ~1.15 Mb in strain 9, and ~1.3 Mb in *D. novamexicana*.

**Figure 4:**
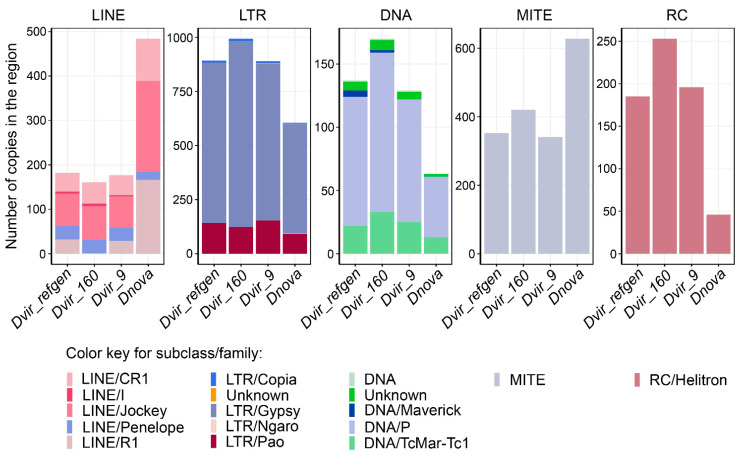
Comparative analysis of transposable element contents in the heterochromatic regions in different *D. virilis* strains and the closely-related species *D. novamexicana*.

Next, we analyzed heterochromatic fragments in terms of the number of TE copies of five different subclasses: LINE (long interspersed nuclear elements) retroelements, LTR (long terminal repeat) retroelements, DNA transposons, DNA/MITE (Miniature inverted repeat transposable elements), and DNA/Rolling circle (RC) elements (Figure 4). As illustrated in Figure 4, TEs composition in the fragments of the different *D. virilis* strains is predominantly similar. Even though there are no active Penelope retroelement copies in the genome of strain 9, we observed an equal number of the diverged Penelope copies in the analyzed fragments of heterochromatin of all *D. virilis* strains studied so far (Figure 4). These copies apparently represent highly diverged remnants of Penelope termed “Omegas” that were shown to be present predominantly in the chromocenter of all virilis group species studied [66,67]. However, there is a slight difference in LINE/R1 and DNA/Maverick copies between *D. virilis* strains (Figure 4). More significant quantitative differences are observed between the two species of the virilis group. Thus, the heterochromatic fragment of *D. virilis* in comparison to *D. novamexicana* is occupied to a higher extent by DNA, DNA/RC, and LTR elements (Figure 4). On the other hand, the *D. novamexicana* fragment contains more LINE and MITE elements (Figure 4). 

Despite the fact that various TE families are present in the considered heterochromatic region for each of the four major subclasses, one specific superfamily is often over-represented: Jockey for LINEs, Gypsy for LTR elements, P for DNA transposons, Helitron for RC elements (Figure 4). Importantly, this observation is in agreement with dominating TE superfamilies estimated in whole genomes even for evolutionary distant species, i.e., *D. melanogaster* and *D. virilis* [68]. 

### 3.5. Pericentromeric Heterochromatin of the Species of Virilis Group Is Evolutionarily Old but Keeps Evolving by Insertion of Young Active Families of Retrotransposons

**Figure 5:**
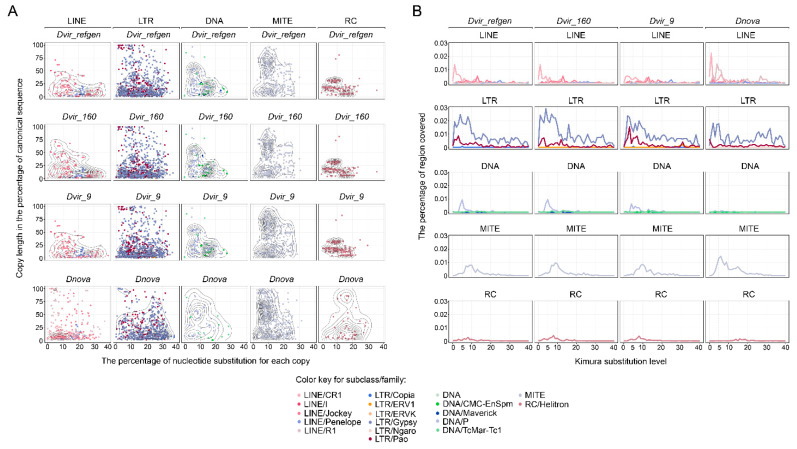
Divergence of transposable elements and the estimated age of X-heterochromatic region in different *D. virilis* strains and closely-related species *D. novamexicana*. (**A**) The relationship of nucleotide substitutions in transposable element copies (the x-axis) to the length of copies (the y-axis). The length of copies is calculated as the percentage from a canonical copy of a transposable element. Each dot represents a single copy of an element. (**B**) Comparison of genetic distances (Kimura 2-distance) of transposable element families in heterochromatic regions.

It is known that the pericentromeric heterochromatin of Drosophila is composed of numerous copies of TEs remnants [1,15]. Indeed, a pericentromeric heterochromatin fragment analyzed in this study includes mostly incomplete and highly diverged TE copies, with exception of a few LINE and LTR retroelements (most of TEs copies are less than 50% of the length of the canonical sequence, with nucleotide divergence more than 10%) (Figure 5A). It is of note that pattern of overall divergence of DNA, DNA/MITE, and DNA/RC elements estimated by both the length of copies and nucleotide substitution level demonstrates the similarity between the *D. virilis* strains and close species, suggesting that these elements had invaded the genome before the speciation of the virilis phylad, which took place about 5 million years ago (Figure 5A) [45]. On the other hand, invasions of LINE and LTR elements into the pericentromeric regions of the virilis group species occurred many times in the evolutionary intervals before and after divergence of *D. virilis* and *D. novamexicana* species. 

To further characterize how TEs activity has been implicated in the evolution of pericentromeric heterochromatin in the species of the virilis group, we constructed the TE landscape divergence plots. As illustrated in Figure 5, pericentromeric regions are mostly occupied by LINE and LTR retroelements that invaded these regions many times during the evolution of virilis phylad species (Figure 5). Interestingly, some of these TE insertions are young (0–2 by Kimura 2-parameter), indicating the ongoing insertion into pericentromeric heterochromatin by these elements (Figure 5B). In agreement with the previous results, TEs dynamics over time indicates that invasions of DNA/MITE and DNA/RC elements are old (peak of the invasion is 8–10 by Kimura substitution level) and apparently describes events that occurred before the divergence of species (Figure 5B). DNA transposons found in the investigated region demonstrate a different pattern of divergence. The most numerous DNA/P family in heterochromatic regions is younger than DNA/MITE and DNA/RC elements (peak of the invasion is 8–10 by Kimura 2-parameter model) and occupied this region in fewer numbers in *D. novamexicana* than *D. virilis* (Figure 5B). Probably DNA transposons had begun to actively invade these pericentromeric regions after the species divergence. 

In spite of the representatives of TE families being highly specific in Drosophila species, the pericentric heterochromatin may include the remnants of ancient invasions that occurred in the ancestral species [1,15]. According to the TE substitution levels, the present state of the pericentric heterochromatin in the species of the virilis group began to form more than 20 million years ago. However, at that time it was mostly occupied by retroelements belonging to LTR/Gypsy, LTR/Pao, and LINE/R1 families (Figure 5B). Over the time interval from 10 to 15 million years ago the pericentromeric region was invaded by MITEs and Helitrons, while the vast majority of other copies of LINE and LTR-containing elements have been invading this chromatin domain starting from 10 million years ago to the present time (Figure 5B). 

### 3.6. Studied Region of Pericentromeric Heterochromatin Has Discrete piRNA-Producing Loci Mostly Targeting Young TE Copies in D. virilis 

Studies of piRNA-generating loci in Drosophila have showed that most piRNAs are produced from long discrete genomic loci termed piRNA-clusters often located in the pericentromeric heterochromatin regions and enriched with diverged copies of TEs [69,70]. Two types of piRNA clusters were described in Drosophila: uni-strand, for which the vast majority of piRNAs maps to one genomic strand, and dual-strand, for which piRNAs originate from both genomic strands [69,70]. We asked whether a comparable region of pericentromeric heterochromatin produces piRNAs and to what extent the piRNAs silence the diverse subclasses of TEs. To analyze the specific genomic regions, we focused on piRNAs in two *D. virilis* strains, 160 and 9, and considered only unique piRNAs (23–29 nt in length) mapped onto the whole genome. 

**Figure 6:**
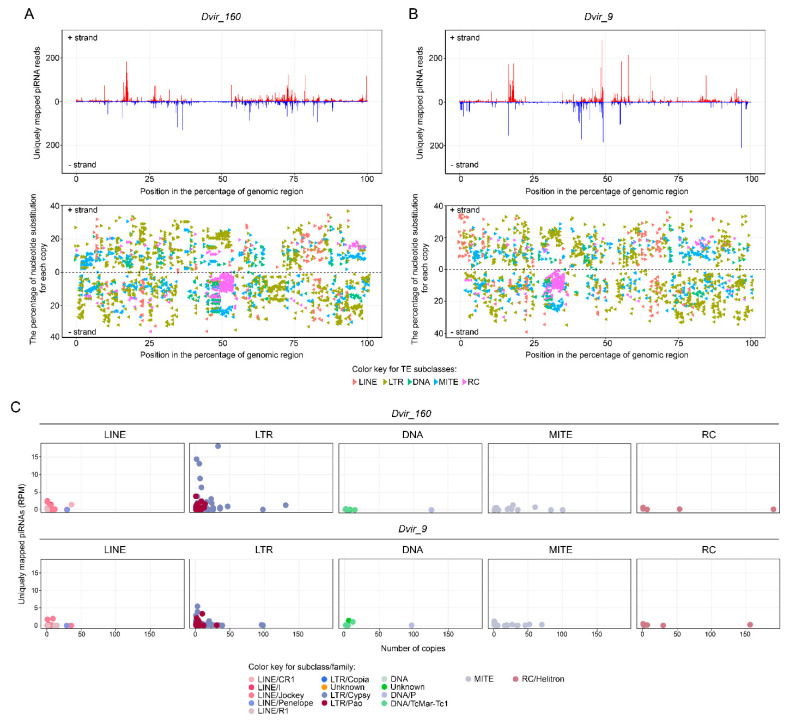
Comparative landscape of piRNA mapping of the studied heterochromatic region in *D. virilis* strains 160 and 9. (**A**,**B**) (Top) The coverage of uniquely mapped piRNAs on [+] and [−] DNA strand of strain 160 and 9, respectively. (Bottom) The content of transposable elements in the considered heterochromatic regions of *D. virilis* strains 160 and 9. Subclasses of transposable elements are indicated by different colors. The orientation of transposable elements is shown relative to [+] and [−] strands. Plots were created by per-base coverage of mapped reads. (**C**) The number of unique piRNAs derived from a genomic region estimated for each transposable element (the y-axis). The number of copies for each transposable element is shown on the x-axis. Each dot represents one transposable element.

The performed analysis shows that the heterochromatic region of the 160 strain has two large distinct loci generating numerous piRNAs (Figure 6A). According to orientation of TEs on [+] and [−] DNA strands, these piRNA-producing loci are predominantly transcribed from both DNA strands, forming dual-strand piRNA clusters (lower part in Figure 6A). The pattern of unique piRNA mappings revealed more discrete piRNA-generating loci in strain 9 with several distinct regions from which the majority of piRNAs originated (Figure 6B). Interestingly, we observed a high concentration of DNA/RC copies in the middle of the region in 160 and to the left from the middle in strain 9 that reach almost 50 Kb in length (lower part of Figure 6A,B). However, none of these regions produce piRNAs in both *D. virilis* strains, suggesting that DNA/RC elements are not active in the genome of *D. virilis*. 

To understand from what specific elements piRNAs are derived, we analyzed a pool of unique piRNAs generated from this region in terms of their targeting of particular elements. The analysis shows that most of piRNAs are derived from LTR/Gypsy, LTR/Pao, LINE/Jockey, LINE/CR1, while only a few piRNAs originated from DNA, MITE and RC elements (Figure 6C and Figure S4). Based on these data combined with the previous results, one may conclude that piRNAs target young elements that are still active in the genome of *D. virilis*. Despite having asymmetric piRNA-clusters in subtelomeric locations, the heterochromatic regions in strains 160 and 9 studied in this work demonstrate consistency in the pattern of piRNA distribution, with a slight difference in the number of piRNAs targeting LTR/Gypsy elements [65]. 

The fact that the pericentromeric heterochromatin is a source for the majority of piRNAs is a well-known phenomenon in Drosophila, however the observed proximity of piRNA-generating loci to protein-coding genes is quite intriguing. As we have shown previously, genes juxtaposed to the pericentromeric heterochromatin in the course of evolution tend to extend their introns due to TE invasion (Figure 1A, Figure 3 and Figure S3). To this end, a number of unique piRNA mappers are derived from TEs residing in intronic regions (Figure S5). It was shown that any sequence embedded in an arbitrary location within an endogenous piRNA-cluster starts to produce piRNAs, becoming part of this piRNA-cluster [71,72]. Given that dual-strand piRNA clusters are transcribed as long precursor transcripts by RNA polymerase II ignoring poly(A) sites of genes due to the activity of Rhino-Deadlock-Cutoff (RDC) complex, how can the parallel expression of protein-coding genes and piRNA from its introns be explained [69,70]? Considering the peculiarities of heterochromatic genes, such as the accumulation of TEs within their introns, one may speculate that heterochromatic genes have become adapted to the heterochromatic environment and are now dependent on heterochromatin-specific proteins that are necessary for the expression of piRNA-clusters [5,69,70,73,74]. Indeed, gene loci relocated to heterochromatin probably retain the transcriptionally active euchromatin-like structure of chromatin capable of efficient transcription in the new location [12,14]. We propose that a euchromatin-like state on promoters and exons and a heterochromatin-like state, with enrichment for H3K9me3 and TEs copies within the introns of genes, are formed simultaneously, allowing both maintenance of gene transcription and piRNA-cluster functioning. However, how this machinery works is the subject for further studies.

## 4. Conclusions

Heterochromatin in *Drosophila* is usually associated with transcriptional silencing. Now it becomes clear that the structural composition of heterochromatin despite its ability to induce gene silencing is far from being inactive. Dozens of essential genes have been embedded into pericentric genome regions due to multiple chromosome rearrangements on the evolutionary timescale. In this study, we investigated the molecular evolution of pericentromeric heterochromatin fragment of chromosome X of *D. virilis* and other Drosophila species. We found that protein-coding genes residing heterochromatic loci are under strong pervasive purifying selection despite their location in the repressive environment mostly formed by different TEs with age more than 20 million years. The evolution in the repetitive context results in the accumulation of TEs within the introns of these genes resulting in a dramatic expansion of gene introns. By surveying structural intra- and interspecific peculiarities of heterochromatin, we found more similar TE composition between *D. virilis* strains than species of the group that differ quantitatively in TEs content. Given that TE remnants of pericentromeric heterochromatin often serve as a major source of piRNA precursors synthesis in *Drosophila* we observed that younger copies of TEs produce piRNA more effectively in terms of the number of mature piRNAs. Interestingly, some piRNA may be derived from TEs residing in intronic regions of actively transcribed heterochromatic genes. Therefore, traditionally being viewed as a highly stable and silent structure of the genome the domains of constitutive heterochromatin exhibit highly dynamic and probably adaptive nature involving complex machinery for its maintenance in an evolving chromosomal architecture. 

## Data Availability

Genomic assembly of *D. virilis* wild type strain 9 was deposited into NCBI under the accession number Bioproject PRJNA687501.

## Supplementary Materials

The following are available online at https://www.mdpi.com/2073-4425/12/2/175/s1, Figure S1: The ratio of nonsynonymous and synonymous substitutions across Diptera species. (A) Results of dN/dS test for *Stim*, *CG8578*, *CG33172*, *Myb*, and *Ranbp16* genes. Estimation was performed relative to the *D. melanogaster* sequence. The calculation for *Stim* gene of *D. novamexicana* as well as for *Stim* and *CG33172* genes of *D. pseudoobscura* is absent due to incomplete annotation of the open reading frames. *Anopheles gambiae* is shown as outgroup. (B) The ratio of nonsynonymous to synonymous substitution rate (ω) on a per-site basis for a multiple coding alignments. Dots represent sites of the coding sequence. Only sites with a *p*-value threshold of < 0.1 are shown; Figure S2: Multiple protein alignment of Leucine-rich repeat of flightless-interacting protein domain of CG8578 protein (A) and WD40 repeat domain of CG33172 protein (B) across selected taxa; Figure S3: Comparative gene organization of *CG33172* (A) and *CG8578* (B) genes among eight *Drosophila* species. White and black regions on the scheme denote untranslated and coding regions, respectively. The grey range between genes indicates homologous regions defined using nucleotide BLAST basing on the percentage of identity not less than 70. The rightwards corner arrow at the beginning of genes indicates the transcriptional start site. Due to the large length of some introns in most *Drosophila* species, all introns are shown not in their real sizes. Numbers beneath the introns reflect their size in nucleotides. Introns that have increased in size by transposable element insertion are highlighted by red bidirectional arrows. The 3′untranslated regions (3′UTR) and 5′UTR are not given for most of the genes due to the absence of their annotations and homology to closely related species; Figure S4: The number of unique piRNAs quantified for each TE subclass derived from a genomic region. Levels of piRNA expression were normalized to the sequencing depth (RPM); Figure S5: Landscape of unique piRNAs mapping on five studied heterochromatic genes of *D. virilis*. Mapping data of piRNAs are not normalized. RNA-seq reads were normalized to the sequencing depth (RPM, reads per million). Repeats and genes are shown in different tracks; Table S1: Accession numbers and genomic coordinates of the studied genes in *Drosophila* species.
