# Supplementary figures and images for: Evolutionary Dynamics of the Pericentromeric Heterochromatin in Drosophila virilis and Related Species
Source: Genes (Basel). 2021 Jan 27;12(2):175. doi: 10.3390/genes12020175 (PMC7911463; doi:10.3390/genes12020175)

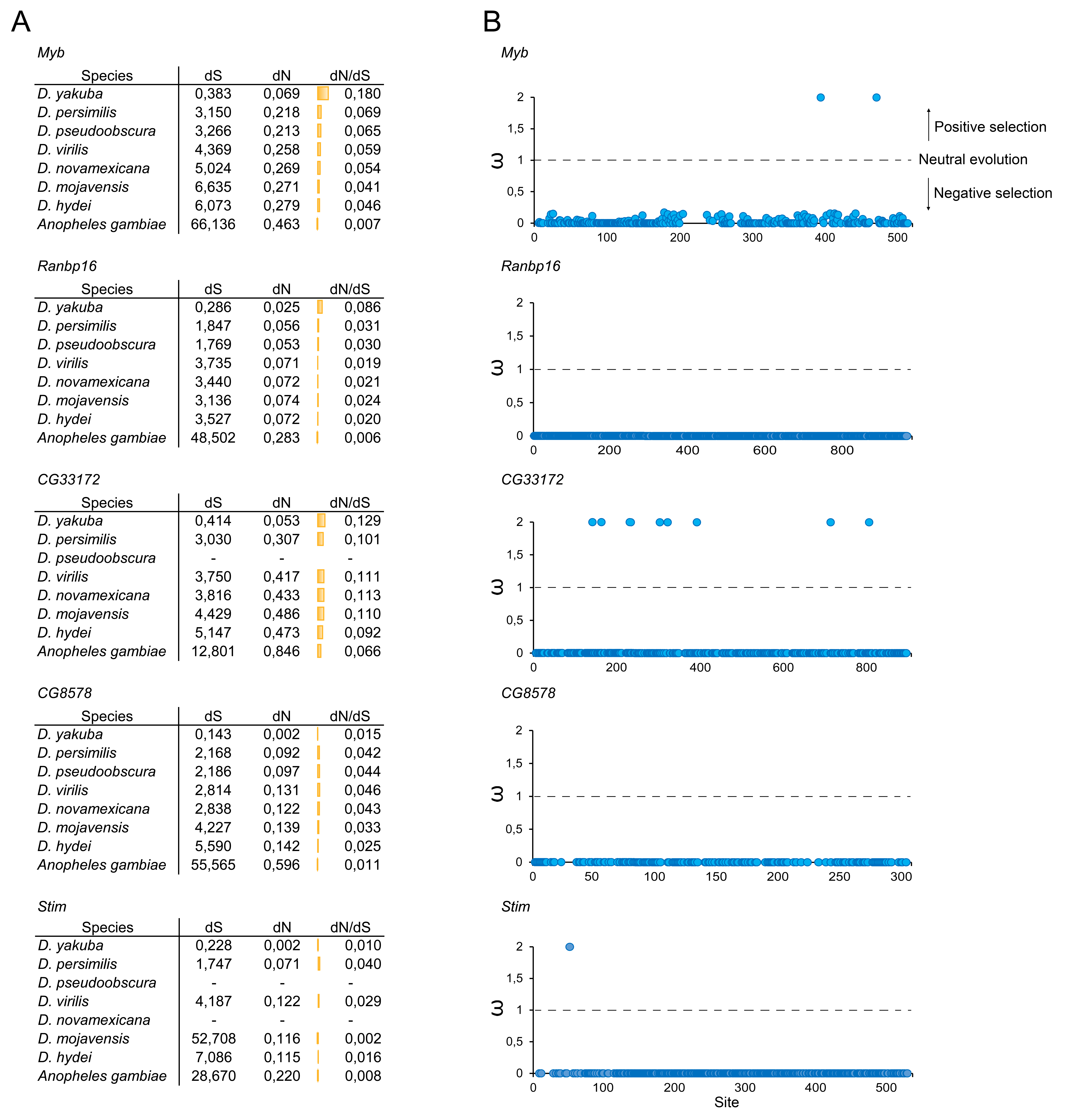

Supplement: Supplementary file 1 [file genes-12-00175-s001.zip › genes-1076739 supplementary/Figure_S1.tif]

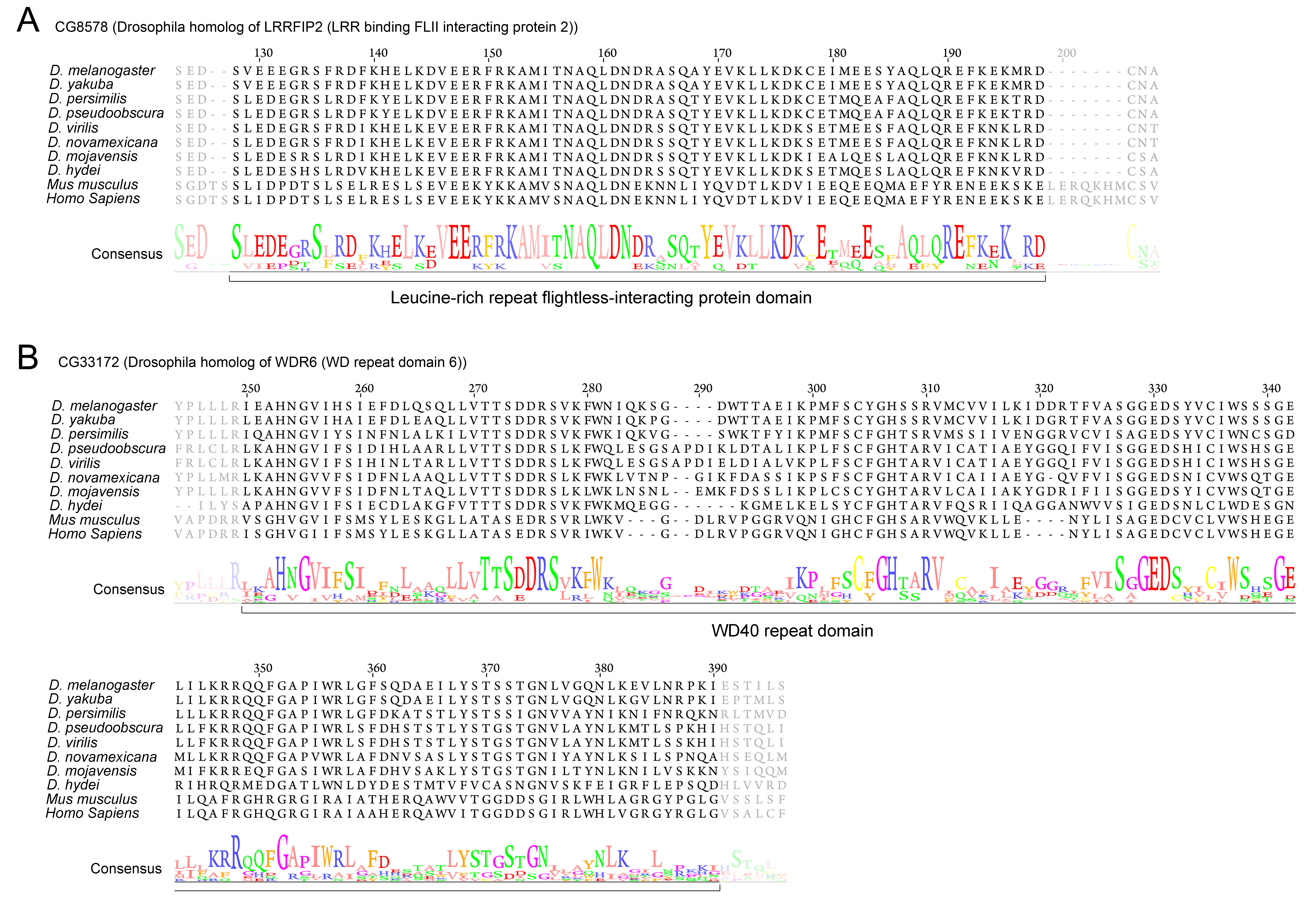

Supplement: Supplementary file 1 [file genes-12-00175-s001.zip › genes-1076739 supplementary/Figure_S2.tif]

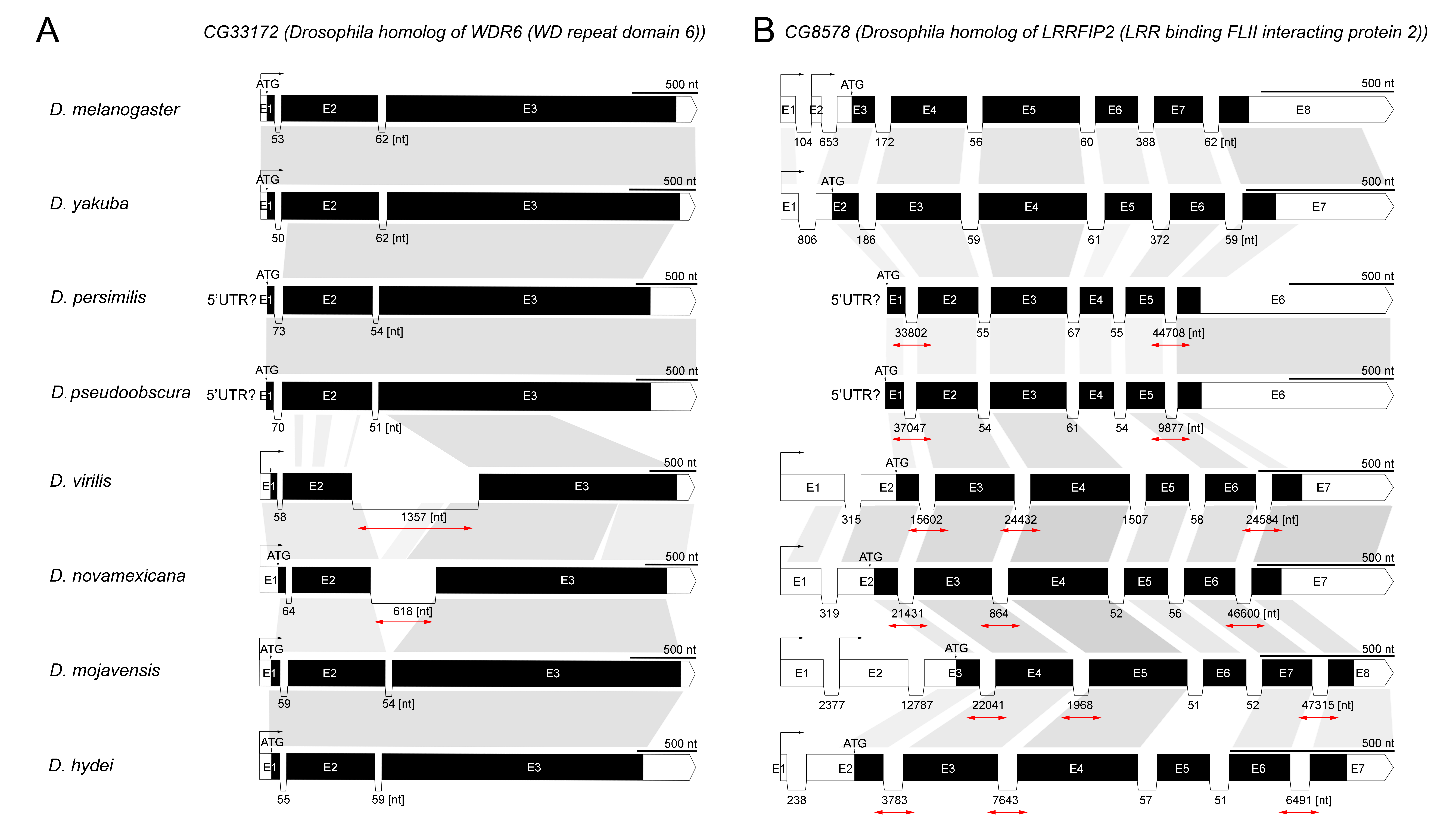

Supplement: Supplementary file 1 [file genes-12-00175-s001.zip › genes-1076739 supplementary/Figure_S3.tif]

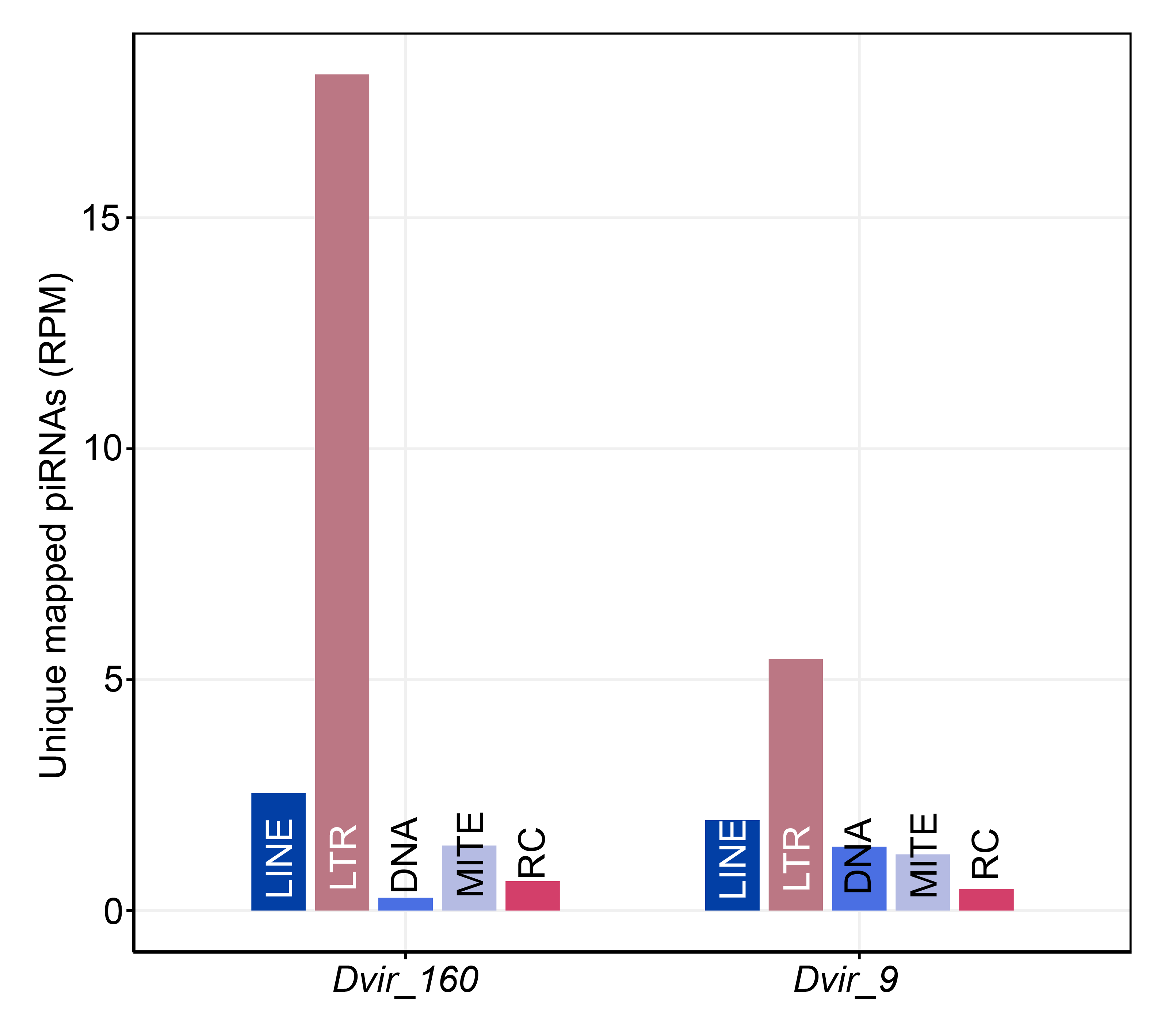

Supplement: Supplementary file 1 [file genes-12-00175-s001.zip › genes-1076739 supplementary/Figure_S4.tif]

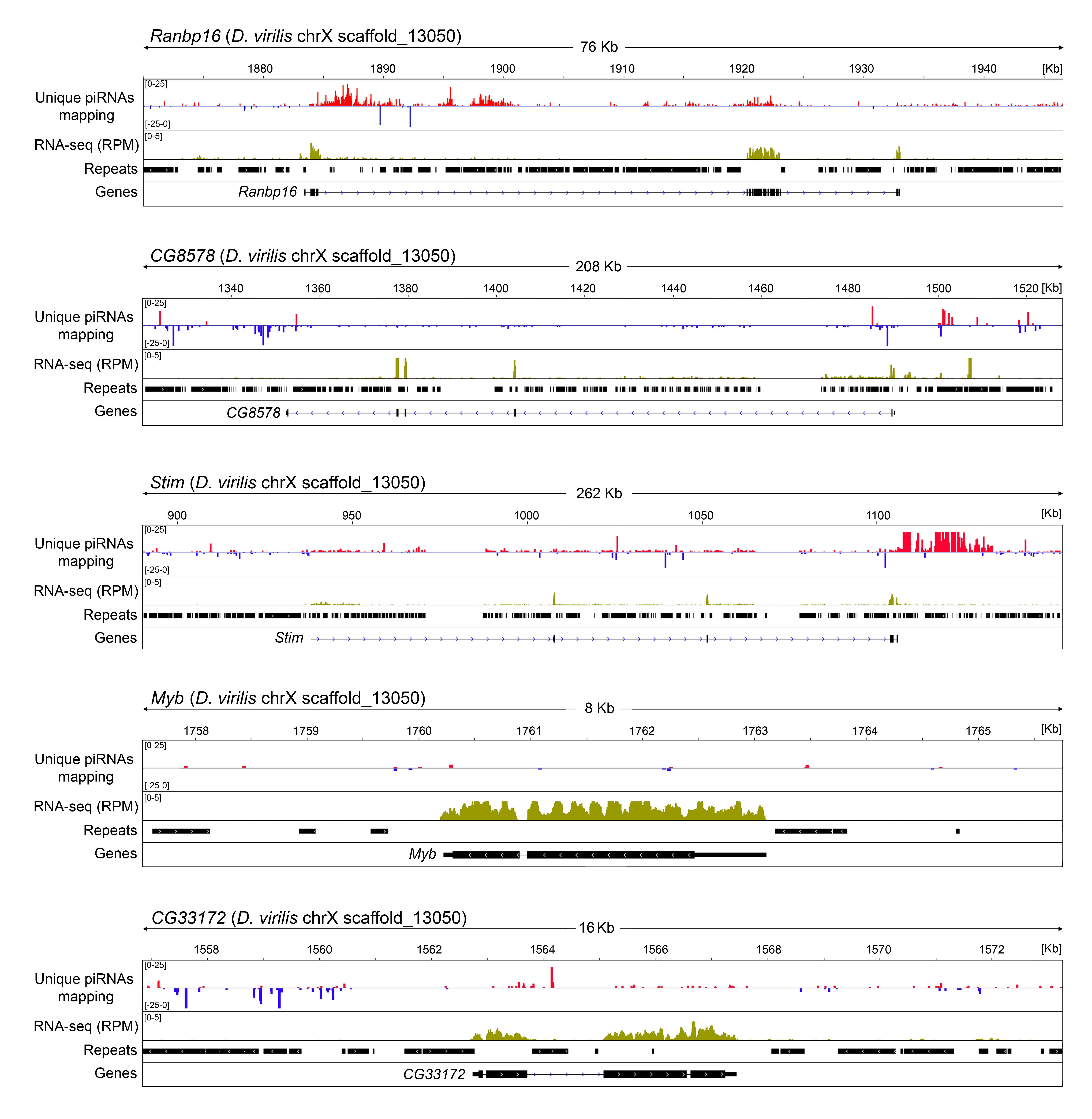

Supplement: Supplementary file 1 [file genes-12-00175-s001.zip › genes-1076739 supplementary/Figure_S5.tif]
